# SIRT2 activates G6PD to enhance NADPH production and promote leukaemia cell proliferation

**DOI:** 10.1038/srep32734

**Published:** 2016-09-02

**Authors:** Shuang-Nian Xu, Tian-Shi Wang, Xi Li, Yi-Ping Wang

**Affiliations:** 1 Department of Haematology, Southwest Hospital, Third Military Medical University, Chongqing 400038, China; 2 Department of Biochemistry and Molecular Cell Biology, Shanghai Key Laboratory for Tumour Microenvironment and Inflammation, Shanghai Jiao Tong University School of Medicine, 280 Chongqing South Rd., Shanghai 200025, China

## Abstract

Like most other types of cancer cells, leukaemia cells undergo metabolic reprogramming to support rapid proliferation through enhancing biosynthetic processes. Pentose phosphate pathway (PPP) plays a pivotal role in meeting the anabolic demands for cancer cells. However, the molecular mechanism by which PPP contributes to leukaemia remains elusive. Here, we report that leukaemia cell proliferation is dependent on the oxidative branch of PPP, in particular the first and rate-limiting enzyme glucose-6-phosphate dehydrogenase (G6PD). Knockdown of *G6PD* reduces NADPH level in acute myeloid leukaemia (AML) cell lines. Exogenous lipid supplements partially restore the proliferation of *G6PD*-depleted cells. Deacetylase SIRT2 promotes NADPH production through deacetylating G6PD at lysine 403 (K403). Activation of G6PD by SIRT2 supports the proliferation and clonogenic activity of leukaemia cells. Chemical inhibitors against SIRT2 suppress G6PD activity, leading to reduced cell proliferation of leukaemia cells, but not normal hematopoietic stem and progenitor cells. Importantly, SIRT2 is overexpressed in clinical AML samples, while K403 acetylation is downregulated and G6PD catalytic activity is increased comparing to that of normal control. Together, our study reveals that acetylation regulation of G6PD is involved in the metabolic reprogramming of AML, and SIRT2 serves as a promising target for further therapeutic investigations.

Dysregulation of metabolic pathways, termed as metabolic reprogramming, is one of characteristic features of cancers^1^. Cancer cells take up and utilize more glucose for glycolysis than oxidative phosphorylation to support rapid cell proliferation (Warburg effect)^2^. The preference towards aerobic glycolysis is critical to provide tumour cells with not only energy, but also multiple building blocks for the biosynthesis of macromolecules, such as proteins, lipids, and nucleic acids *etc*^3^.

Acute myeloid leukaemia (AML) is the most common type of malignant myeloid disorder in adults^4^,^5^. Recent evidence revealed that leukaemia cells exhibited an increased dependence on aerobic glycolysis^6^,^7^. Mouse model studies further demonstrated that enhanced glucose uptake accelerated leukemogenesis *in vivo*^8^,^9^,^10^. Besides, glycolysis inhibition resulted in growth arrest or cell death of AML cells, and sensitized leukaemia cells to chemotherapeutic agents^7^,^11^. Notably, pentose phosphate pathway (PPP), a shunt for glycolysis, was also frequently altered in AML. Glucose turnover in leukaemia cells was possibly enhanced via PPP pathway rather than glycolysis^12^,^13^,^14^,^15^, indicating the implication of PPP pathway in metabolic reprogramming of leukaemia.

Glucose is converted into glucose-6-phosophate (G6P) upon entering into the cell. Afterward, G6P is either utilized in glycolysis for catabolism, or shunted towards pentose phosphate pathway. Based on the catalytic properties, PPP can be divided into oxidative and non-oxidative phases. In the oxidative branch, glucose-6-phosphate dehydrogenase (G6PD) and 6-phosphogluconate dehydrogenase (PGD) catalyse sequential dehydrogenation reactions of G6P, yielding five-carbon sugars (ribose) and nicotinamide adenine dinucleotide phosphate (NAPDH). In the non-oxidative phase, transketolase (TKT) and transaldolase (TALDO1) converted/recycled five-carbon sugars into glycolytic intermediates. Ribose is an essential precursor for nucleotide synthesis^16^, while NADPH functions as a reducing power for fatty acid synthesis and anti-oxidant processes. By producing theses two key intermediates (ribose and NADPH), PPP pathway plays a pivotal role in meeting the anabolic demands for cancer cells^17^,^18^,^19^,^20^,^21^.

G6PD is the first and rate-limiting enzyme of PPP. G6PD deficiency is one of the most common enzyme defects in humans, affecting 400 million people worldwide^22^. The lack of G6PD results in shortage of NADPH and deficiency of red blood cells (RBCs) in scavenging ROS, clinically characterized by hemolysis and anaemia^23^. Previously, we found that G6PD was post-translationally modified by lysine acetylation. SIRT2 deacetylated G6PD at lysine 403 (K403) and enhanced its activity in response to oxidative stress, protecting RBCs from oxidative damage^24^. In addition, recent studies demonstrated that G6PD was implicated in the development of multiple cancers, including breast cancer, liver cancer, leukaemia *etc*^25^. Leukaemia cells expressed high level of G6PD, even in G6PD-deficient subjects^26^. In chronic myelomonocytic leukaemia (CMML), overexpression of *G6PD* mRNA associated with poor diagnosis^27^. These observations indicate a potential link between G6PD deregulation and leukaemia development. However, the role of G6PD in metabolic reprogramming of leukaemia remains unclear. This study investigates the functional significance of PPP pathway, especially G6PD, in leukaemia development.

## Results

### Oxidative PPP is essential for the proliferation of leukaemia cells

**Figure 1:**
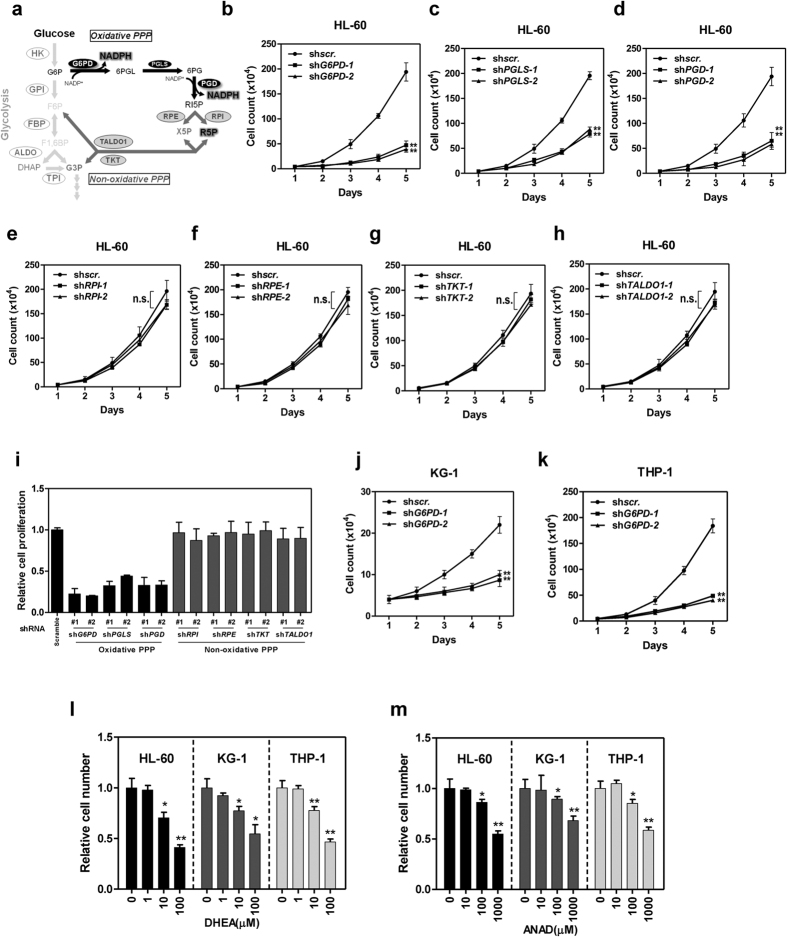
G6PD is essential for the proliferation of leukaemia cells. (**a**) Schematic overview of pentose phosphate pathway. Enzymes for individual chemical reactions are labelled as ovals and denoted next to the arrows connecting two metabolites. Metabolites and enzymes in oxidative PPP are colored in black, non-oxidative PPP in dark grey. G6P, glucose 6-phosphate; F6P, fructose 6-phosphate; F1,6BP, fructose 1,6-bisphosphate; DHAP, dihydroxyacetone phosphate; G3P, glyceraldehydes 3-phosphate; 6PGL, 6-phosphogluconolactone; 6PG, 6-phosphogluconate; R5P, ribulose 5-phosphate; X5P, xylulose 5-phosphate. (**b**–**h**) The proliferation curve of HL-60 cells expressing a control shRNA (shscr.) or shRNAs against *G6PD* (**b**), *PGLS* (**c**), *PGD* (**d**), *RPI* (**e**), *RPE* (**f**), *TKT* (**g**), or *TALDO1* (**h**) was determined by cell counting. (**i**) HL-60 cells stably expressing control shRNA (scramble) or shRNAs targeting genes in PPP pathway as indicated were grown for 5 days, relative cell growth was determined by CCK8 assay. (**j**–**k**) The proliferation of KG-1 (**j**) and THP-1 (**k**) cells stably expressing control shRNA (shscr.) or shRNAs against *G6PD* were determined by cell counting. (**l**,**m**) HL-60, KG-1 and THP-1 cells were grown for 5 days with or without treatment of increasing concentrations of DHEA (**l**) or ANAD (**m**). Relative cell growth was determined by cell counting. Error bars represent mean ± SD from three replicates of each sample (*p < 0.05, **p < 0.01, n.s. = not significant for the indicated comparison).

PPP pathway sustains rapid cell growth by providing NADPH and pentose to biosynthetic processes (Fig. 1a). To dissect the contribution of PPP to leukaemia, we constructed a shRNA library targeting PPP enzymes and tested the dependence of leukaemia cell proliferation on these enzymes. Interestingly, depletion of enzymes in oxidative PPP, i.e. *G6PD*, *PGLS* (6-phosphogluconolactonase), and *PGD*, dramatically abrogated the proliferation of HL-60, a human promyelocytic leukaemia cell line (Fig. 1b–d and s1a). Contrarily, knockdown of non-oxidative PPP enzymes, including *RPE* (ribulose 5-phosphate 3-epimerase), *RPI* (ribulose 5-phosphate isomerase), *TALDO1* (transaldolase), and *TKT* (transketolase), had negligible effects on cell proliferation (Fig. 1e–h and s1a). Accordingly, CCK-8 assay also demonstrated that oxidative PPP, but not non-oxidative PPP, is necessary for the proliferation of leukaemia cells (Fig. 1i). In support of these observations, cell growth of another two AML cell lines with different FAB subtypes (THP-1 and KG-1) was remarkably suppressed upon shRNA-induced *G6PD* knockdown (Supplementary Table 2 and Fig. 1j,k). Moreover, G6PD inhibitors, i.e. dehydroepiandrosterone (DHEA) and 6-aminonicotinamide (ANAD), significantly decreased the proliferation of HL-60, KG-1, and THP-1 cells in a dose-dependent manner (Fig. 1l,m). Together, these data demonstrate that leukaemia cell proliferation is dependent on the oxidative branch of PPP, in particular G6PD, across different subtypes.

### G6PD maintains NADPH level in leukaemia cells

**Figure 2:**
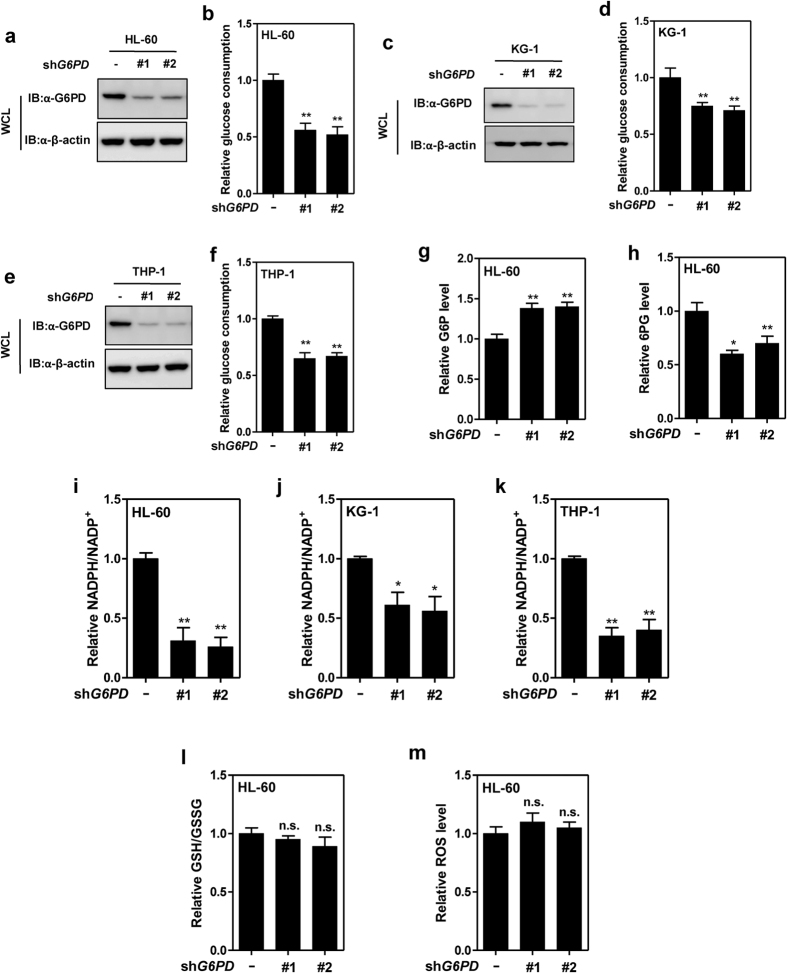
G6PD maintains NADPH level in leukaemia cells. (**a**–**f**) Knockdown efficiencies of shRNAs targeting G6PD in HL-60 (**a**), KG-1 (**c**), and THP-1 (**e**) cells was determined by western blotting. Relative glucose consumptions of HL-60 (**b**), KG-1 (**d**), and THP-1 (**f**) stable cells were determined. (**g**,**h**) Relative concentrations of G6P (glucose 6-phosphate) (**g**) and 6PG (6-phosphpogluconate) (**h**) in control or G6PD-knockdown HL-60 cells were determined. (**i**–**k**) Relative NADPH/NADP^+^ ratios in control or G6PD-knockdown HL-60 (**i**), KG-1 (**j**), and THP-1 (**k**) cells were determined. (**l**,**m**) Relative GSH/GSSG ratio (I) and ROS level (**m**) in control or G6PD-knockdown HL-60 cells were determined. Error bars represent mean ± SD from three replicates of each sample (*p < 0.05, **p < 0.01, n.s. = not significant for the indicated comparison).

Next, we investigated metabolic alterations caused by *G6PD* knockdown. G6PD converts G6P and coenzyme NADP^+^ to 6PG and NADPH (Fig. 1a). Depletion of *G6PD* significantly reduced glucose consumption of HL-60, KG-1 and THP-1 cells (Fig. 2a–f). In accordance, knockdown of *G6PD* resulted in 1.4-fold accumulation of G6P (p = 0.015) and a 30% reduction of 6PG (p = 0.032) in HL-60 (Fig. 2g,h). Cellular NADPH/NADP^+^ ratio was significantly decreased by *G6PD* depletion in HL-60, KG-1 and THP-1 cells (Fig. 2i–k). These results suggest that G6PD is essential for cellular NADPH production in leukaemia cells.

NADPH can be utilized in the regeneration of reduced glutathione (GSH), which detoxifies reactive oxygen species (ROS). Interestingly, depletion of *G6PD* altered neither the ratio of reduced to oxidized glutathione (GSH/GSSG), nor cellular ROS level of HL-60 (Fig. 2l,m). These results indicate that ROS scavenging was not impaired by the decline of NADPH supply in *G6PD*-knockdown cells.

### G6PD-deficient cells exhibit an increased demand for lipids

**Figure 3:**
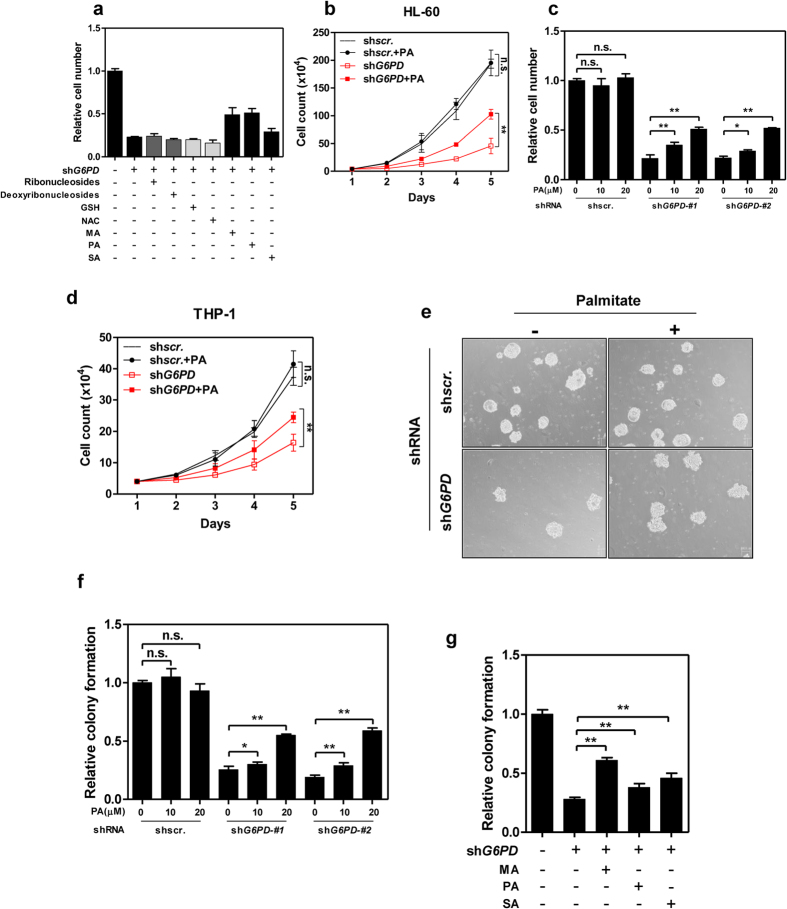
Supplementation of lipids rescues proliferation and colony formation of *G6PD*-knockdown cells. (**a**) Control or *G6PD*-knockdown stable HL-60 cells were grown for 5 days. *G6PD*-depleted cells were either untreated or supplemented with ribonucleosides, deoxyribonucleosides, 5mM reducing agents (GSH or NAC), or 20 μM lipids (MA, myristic acid; PA, palmitic acid; SA, stearic acid) as indicated. Relative cell growth was determined by cell counting. (**b**) Control (shscr.) and *G6PD*-knockdown stable HL-60 cells were treated with or without palmitic acid (PA), relative cell proliferation was determined by cell counting. (**c**) Control or *G6PD*-knockdown stable HL-60 cells were treated with increasing concentrations of palmitic acid as indicated and grown for 5 days. Relative cell growth was determined by cell counting. (**d**) Control (shscr.) and *G6PD*-knockdown stable THP-1 cells were treated with or without palmitic acid (PA), relative cell proliferation was determined by cell counting. (**e**) Control and *G6PD*-knockdown stable HL-60 cells were treated with or without palmitic acid. Colony formation of was determined. Shown are representative images (Bar = 100 μm). (**f**) Control or *G6PD*-knockdown stable HL-60 cells were supplemented with increasing concentrations of palmitic acid as indicated. Relative colony formation was determined. (**g**) Control or G6PD-knockdown stable HL-60 cells were supplemented with or without different lipids (20 μM) as indicated. Relative colony formation was determined. Error bars represent mean ± SD from three replicates of each sample (*p < 0.05, **p < 0.01, n.s. = not significant for the indicated comparison).

Alternatively, the NADPH produced by G6PD may support biosynthesis of macromolecules in leukaemia cells. If so, exogenous nutrient supplements may rescue the growth defect of *G6PD*-depleting cells. To identify potential biosynthetic process(es) affected by G6PD depletion, we added different supplements, including nucleosides and lipids, into the culture medium of *G6PD*-knockdown cells. In line with our earlier findings, reducing agents (GSH and N-acetyl cysteine) were incapable of restoring the proliferation of *G6PD*-depleting cells (Fig. 3a). Similarly, ribonucleosides or deoxyribonucleosides failed to restore the growth of *G6PD*-knockdown cells (Fig. 3a). However, supplementation of lipids, such as myristic acid (MA), palmitic acid (PA), or stearic acid (SA), partially rescued the growth defect of *G6PD*-knockdown cells (Fig. 3a). Of note, PA partially restored the proliferation of *G6PD*-knockdown cells, but not control HL-60 or THP-1 cells (Fig. 3b–d). These results indicate that the NADPH produced by G6PD is possibly utilized in lipogenic reactions of leukaemia cells, explaining increased lipid requirements of *G6PD*-deficient AML cells.

We further investigated the effect of PA on clongenic ability of leukaemia cells. Knockdown of *G6PD* severely reduced the colony formation of HL-60 cells, which was partly rescued by PA supplementation in a dose dependent manner (Fig. 3e,f). Meanwhile, clongenic activity of control cells remained unaltered by PA treatment (Fig. 3e,f). Interestingly, MA and SA also partially restored the colony formation of *G6PD*-deficient cells (Fig. 3g). Together, G6PD activity is essential for both cell proliferation and colony formation of leukaemia cells, presumably through supporting *de novo* lipogenesis.

### SIRT2-mediated deacetylation of G6PD promotes NADPH production

**Figure 4:**
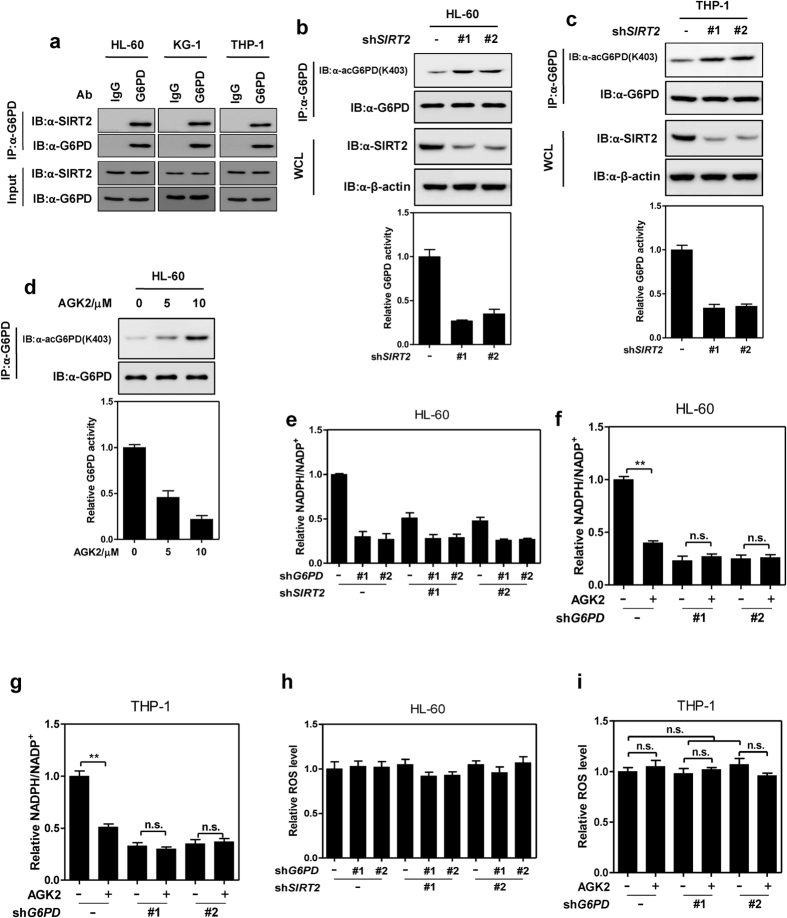
Deacetylation of G6PD by SIRT2 promotes NADPH production. (**a**) Endogenous G6PD protein was immunoprecipitated from human AML cell lines (HL-60, KG-1 and THP-1), followed by western blotting to detect SIRT2. Normal rabbit IgG was included as a negative control. (**b**,**c**) Endogenous G6PD protein was immunoprecipitated from control or *SIRT2*-knockdown stable HL-60 (**b**) or THP-1 (**c**) cells, followed by western blotting to detect K403 acetylation [acG6PD (K403)]. Catalytic activity of endogenous G6PD was determined. WCL denotes whole cell lysate (**d**) HL-60 cells were treated with increasing doses of AGK2 as indicated for 6hrs. K403 acetylation and catalytic activity of immunopurified endogenous G6PD was determined. (**e**) Relative NADPH/NADP^+^ ratio in HL-60 cells stably expressing shRNA against *G6PD* and/or *SIRT2* was determined. (**f**,**g**) Control or G6PD-knockdown stable HL-60 (**f**) and THP-1 (**g**) cells were treated with or without 5 μM AGK2 for 6hrs. Relative NADPH/NADP^+^ ratio were determined. (**h**) Relative ROS level in HL-60 cells stably expressing shRNA against *G6PD* and/or *SIRT2* was determined. (**i**) Control or G6PD-knockdown stable THP-1 cells were treated with or without 5 μM AGK2 for 6 hrs. Relative ROS level was determined. Error bars represent mean ± SD from three replicates of each sample (**p < 0.01, n.s. = not significant for the indicated comparison).

The dependence of leukaemia cell on G6PD implies that suppression of oxidative PPP, in particular G6PD, may serve as a promising strategy to inhibit leukaemia. However, targeting PPP remains challenging due to the lack of specific G6PD inhibitors. Previously, we found that G6PD was post-translationally modified by lysine acetylation. SIRT2 deacetylated G6PD at lysine 403 (K403) and activated its activity^24^. Thus, inhibition of SIRT2 would be an alternative approach to suppress G6PD and oxidative PPP. To this end, we examined the physiological significance of the interaction between SIRT2 and G6PD in leukaemia cells. Endogenous immunoprecipitation assay demonstrated that the physical interaction between G6PD and SIRT2 was readily detectable in HL-60, KG-1, and THP-1 cells (Fig. 4a). shRNA-induced knockdown of *SIRT2* elevated G6PD K403 acetylation and remarkably decreased G6PD activity in HL-60 and THP-1 cells (Fig. 4b,c). Moreover, SIRT2-specific inhibitor AGK2 increased G6PD acetylation and reduced its catalytic activity in a dose-dependent manner (Fig. 4d). These data clearly indicate that G6PD is deacetylated and activated by SIRT2 in leukaemia cells.

Next, we investigated the role of SIRT2-mediated deacetylation and activation of G6PD in glucose utilization and NADPH production. Glucose uptake of HL-60 was inhibited upon knockdown of *SIRT2* or *G6PD*, while depletion of *SIRT2* did not led to further reduction of glucose consumption in *G6PD*-knockdown cells (Fig. s2a,b). Additionally, NADPH/NADP^+^ ratio was decreased by two-fold in *SIRT2*-knockdown cells, while depleting SIRT2 exhibited no effect on NADPH level in *G6PD*-knockdown HL-60 cells (Fig. 4e). AGK2 treatment reduced the NADPH/NADP^+^ ratio of control cells, but not *G6PD*-knockdown HL-60 or THP-1 cells (Fig. 4f,g). Of note, inhibiting SIRT2 using shRNA or chemical inhibitor AGK2 displayed no effect on cellular redox state of HL-60 or THP-1 cells (Fig. 4h,i). These results suggest that SIRT2 maintains NADPH level in a manner dependent on G6PD.

### Inhibition of SIRT2 reduces leukaemia cell proliferation

**Figure 5:**
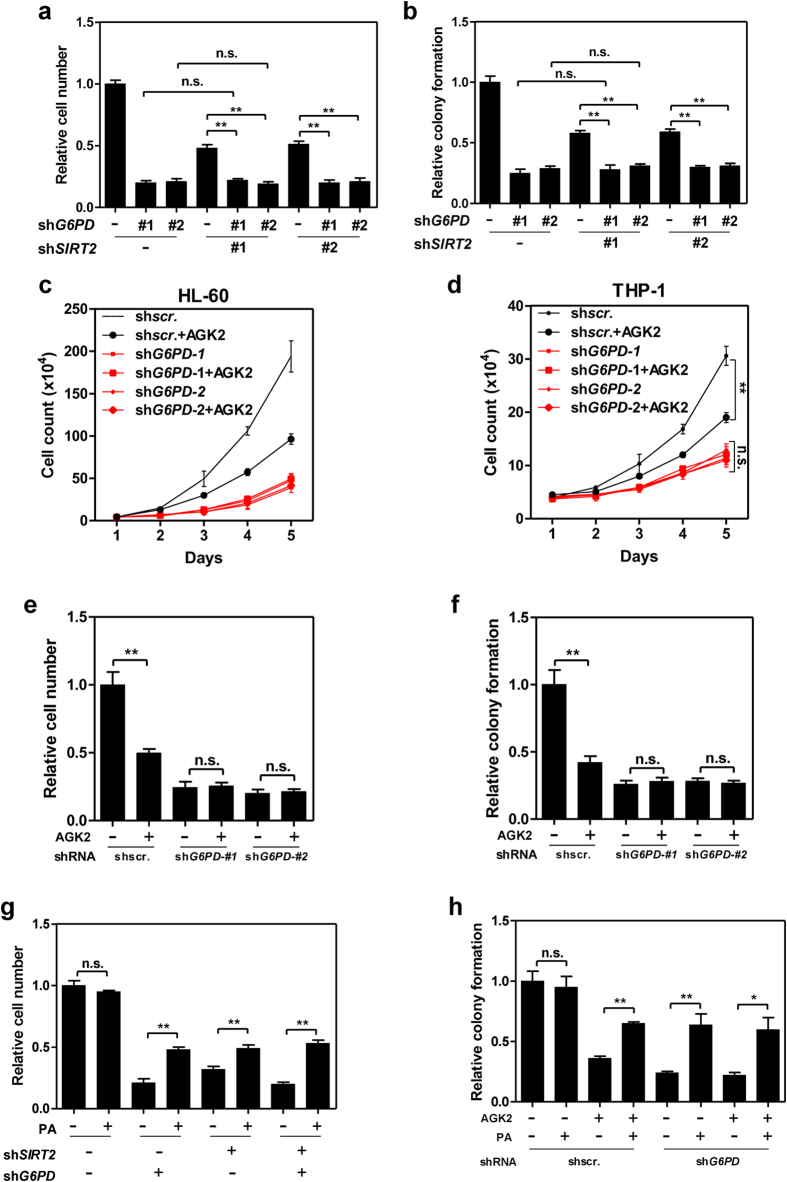
Inhibiting the deacetylation of G6PD reduces leukaemia cell proliferation. (**a**) Stable *G6PD*- and/or *SIRT2*-knockdown HL-60 cells as indicated were grown for 5 days, relative cell growth was determined by cell counting. (**b**) Colony formation of *G6PD*- and/or *SIRT2*-knockdown stable HL-60 cells was determined. (**c**,**d**) Control or *G6PD*-knockdown HL-60 (**c**) and THP-1 (**d**) cells were treated with or without AGK2. Cell proliferation was determined by cell counting. (**e**,**f**) Relative cell growth (**e**) and colony formation (**f**) of control or G6PD-knockdown HL-60 cells with indicated treatment was determined by CCK-8 assay and clonogenic assay, respectively. (**g**) *G6PD*- and/or *SIRT2*-knockdown stable HL-60 cells were supplemented with or without PA, relative cell growth was determined by cell counting after cells were grown for 5 days. (**h**) Control or *G6PD*-knockdown HL-60 cells were treated as indicated, colony formation was determined. Error bars represent mean ± SD from three replicates of each sample (*p < 0.05, **p < 0.01, n.s. = not significant for the indicated comparison).

We next explored the functional role of acetylation regulation of G6PD by SIRT2 in leukaemia cell proliferation. Depletion of *SIRT2* reduced both cell growth and clongenic ability of control HL-60 cells, but not *G6PD*-knockdown cells (Fig. 5a,b). Consistently, AGK2 treatment failed to suppress the proliferation and colony formation of *G6PD*-knockdown HL-60 and THP-1 cells (Fig. 5c–f). Interestingly, PA supplementation partially restored the proliferation and colony formation of *SIRT2*- and/or *G6PD*-knockdown cells, but not control HL-60 cells (Fig. 5g). Suppression of cell growth and colony formation by AGK2 was partly rescued by PA treatment (Fig. 5h). These data demonstrate that the contribution of SIRT2 to leukaemia cell proliferation and colony formation is dependent on G6PD.

### Small molecule inhibitors of SIRT2 decreases G6PD activity and suppresses leukaemia cell proliferation

**Figure 6:**
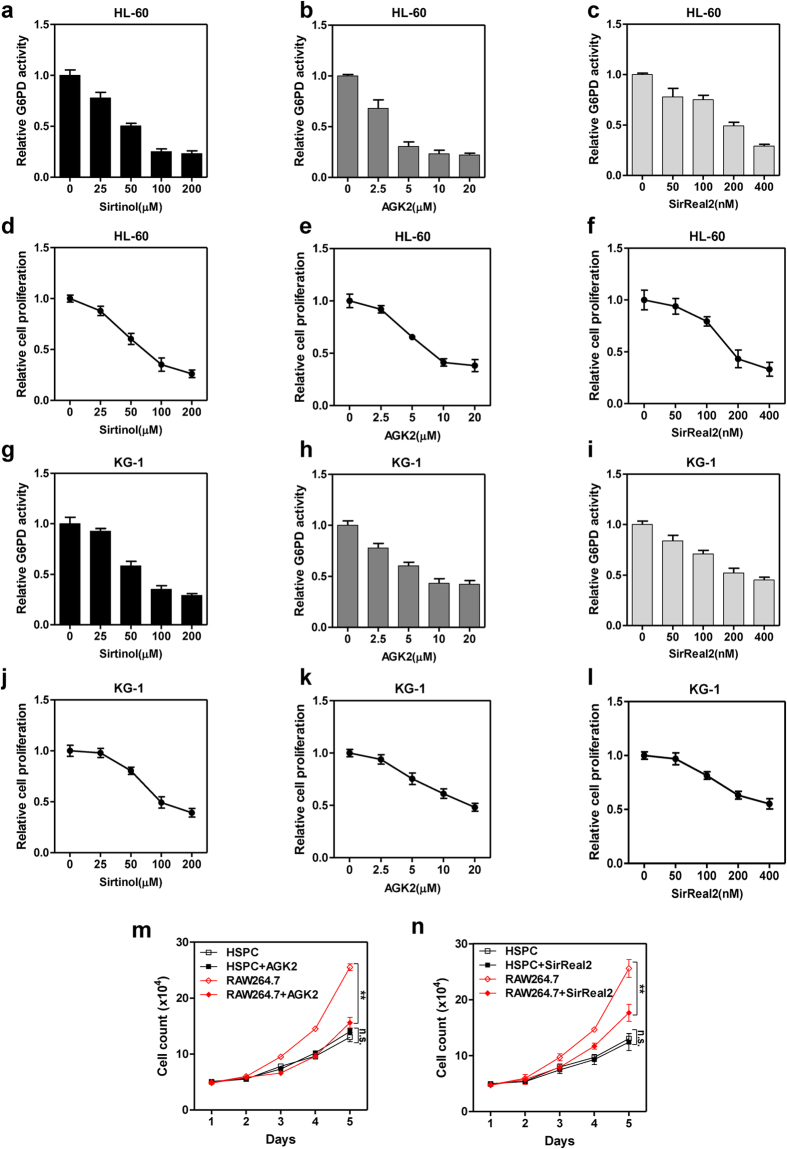
Small molecule inhibitors of SIRT2 decrease G6PD activity and suppress leukaemia cells. (**a**–**c**) HL-60 cells were treated with increasing doses of SIRT2 inhibitors, sirtinol (**a**), AGK2 (**b**) and SirReal2 (**c**), for 6 hours as indicated, G6PD activity was determined. (**d**–**f**) HL-60 cells were treated with increasing doses of SIRT2 inhibitors, sirtinol (**d**), AGK2 (**e**) and SirReal2 (**f**), relative cell growth was determined by cell counting after grown for 5 days. (**g**–**i**) KG-1 cells were treated with increasing doses of SIRT2 inhibitors, sirtinol (**g**), AGK2 (**h**) and SirReal2 (**i**), for 6 hours as indicated, G6PD activity was determined. (**j**–**l**) KG-1 cells were treated with increasing doses of SIRT2 inhibitors, sirtinol (**j**), AGK2 (**k**) and SirReal2 (**l**), relative cell growth was determined by cell counting after grown for 5 days. (**m**,**n**) Mouse hematopoietic stem and progenitor cells (HSPC) and RAW264.7 cells were treated with 5 μM AGK2 (**m**) or 150 nM SirReal2 (**n**) as indicated and grown for 5 days, cell proliferation curve was determined by cell counting. Error bars represent mean ± SD from three replicates of each sample (**p < 0.01, n.s. = not significant for the indicated comparison).

The observation that SIRT2-induced activation of G6PD contributes to leukaemia indicates that inhibiting SIRT2 with small molecule inhibitors may serve as a substitutive strategy to suppress G6PD. Recently, a spectrum of chemical inhibitors against SIRT2 has been developed, from which we utilized sirtinol, AGK2, and SirReal2 to evaluate their anti-proliferative effects (Supplementary Table 3). Interestingly, SIRT2 inhibitors dose-dependently decreased G6PD activity (Fig. 6a–c), and resulted in declined proliferation of HL-60 cells (Fig. 6d–f). SIRT2 inhibitors also suppressed G6PD activity (Fig. 6g–i) and cell proliferation (Fig. 6j–l) in KG-1 cells. To test the preference of SIRT2 inhibitors, we isolated normal hematopoietic stem and progenitor cells (HSPCs) and found that SIRT2-specific inhibitors (AGK2 and SirReal2) selectively decreased the proliferation of mouse leukaemia cell RAW264.7, but not HSPCs (Fig. 6m,n). These data suggest that SIRT2 inhibitors efficiently inactivate G6PD, and preferentially inhibit the proliferation of leukaemia cells.

### K403 acetylation of G6PD is downregulated in AML

**Figure 7:**
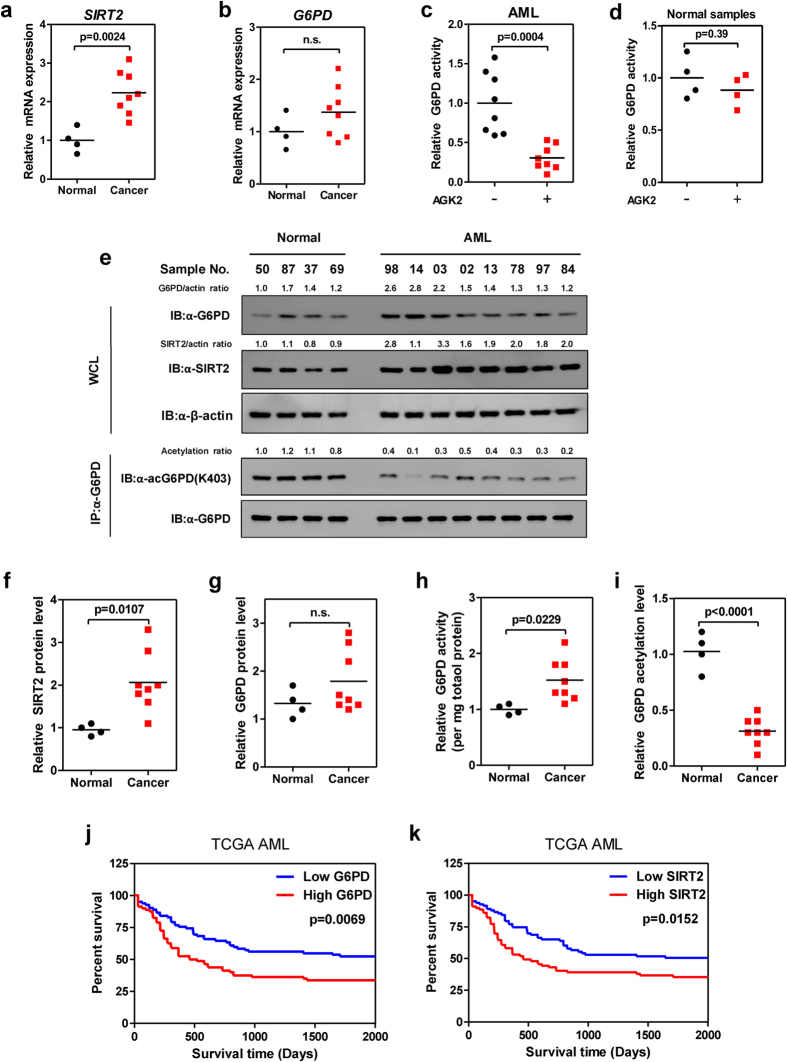
Acetylation at K403 of G6PD is downregulated in AML. (**a**,**b**) Relative mRNA expression of *SIRT2* (A) and *G6PD* (B) in control and AML samples was determined by qRT-PCR. (**c**,**d**) Leukaemia (**c**) or normal (**d**) cells from 4 healthy donors and 8 clinical AML patients were treated with or without 5 μM AGK2 for 6hrs, G6PD activity was determine. (**e**–**i**) Clinical AML samples and normal controls were lysed. Protein expression was determined by western blotting as indicated (**e**), SIRT2 (**f**) and G6PD (**g**) expression were quantified and normalized to β-actin (n.s. = not significant). G6PD activities were determined and normalized against G6PD protein (**h**). K403 acetylation level of immunopurified G6PD was quantified. Acetylation ratio was normalized against G6PD protein (**i**). (**j**,**k**) Kaplan-Meier survival curves for TCGA AML study. The patients (n = 173) were stratified by median G6PD (**j**) and SIRT2 (**k**) levels (‘high’ is greater than median, ‘low’ is less than or equal to median). *p < 0.05, **p < 0.01, n.s. = not significant for the indicated comparison.

We further explored the pathological relevance of SIRT2-induced G6PD activation in clinical samples of AML. We collected mononuclear cells from bone marrows of 8 AML patients and 4 healthy donors (Supplementary Table 4). mRNA expression analysis showed that AML cells expressed higher level of *SIRT2* than normal controls (p = 0.0024) (Fig. 7a), while no significant difference was observed in G6PD *mRNA* expression (Fig. 7b). Notably, AGK2 treatment strongly inhibited G6PD activity in AML cells (p = 0.0004), but not normal samples (Fig. 7c,d). Direct immunoblotting assay demonstrated that SIRT2 protein was significantly increased (p = 0.0107), while G6PD was mildly overexpressed in AML samples (Fig. 7e–g). Of note, G6PD enzymes in AML samples were more active than that from normal tissues (p = 0.0229) (Fig. 7h). Accordingly, K403 acetylation of G6PD was reduced in AML cells (p < 0.0001) (Fig. 7i). These data indicate that G6PD K403 acetylation is downregulated in AML. We further carried out a survival analysis by using TCGA AML cohort^28^. In line with our findings, high expression of G6PD, but not the other enzymes in PPP, significantly associated with poor survival rate (p = 0.0069) (Fig. 7j and Fig. s3a,b). Reduced survival rate was also observed in AML cases overexpressing SIRT2 (p = 0.0152) (Fig. 7k). These data strongly support that acetylation regulation of G6PD by SIRT2 is involved in the development of AML.

## Discussion

**Figure 8:**
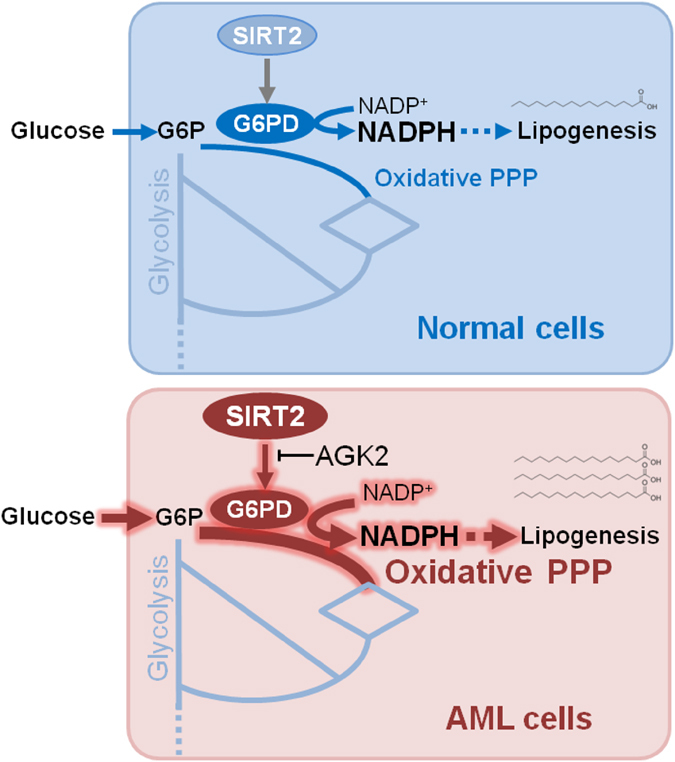
The model for SIRT2-mediated G6PD acetylation regulation in AML.

Cancer metabolism is reprogrammed to enhance the biosynthesis of diverse macromolecules, fulfilling requirements for rapid proliferation. In this study, we investigated the dysregulation of PPP pathway in AML. We demonstrated that proliferation of leukaemia cells was dependent on the oxidative branch of PPP, in particular G6PD. *G6PD*-knockdown cells had decreased NADPH/NADP^+^ ratio, and exhibited an enhanced demand for lipids, indicating that the NADPH produced by G6PD was necessary for *de novo* lipid synthesis. In addition, SIRT2 deacetylated and activated G6PD to maintain intracellular NADPH level. Chemical inhibitors against SIRT2 effectively impaired G6PD activity and suppressed the proliferation of leukaemia cells. Of note, we found that SIRT2 was overexpressed, with corresponding low K403 acetylation levels of G6PD, in clinical AML samples. High SIRT2 expression correlated with poor overall survival rate in AML patients. Taken together, our study reveals that deacetylation of G6PD by SIRT2 sustains NADPH production and promotes leukaemia cell proliferation (Fig. 8).

Although G6PD serves as a potential target to suppress leukaemia, ANAD is not amenable to *in vivo* use because of its toxic side effects^29^. DHEA is a naturally occurring hormone with pleiotropic effects that may be unrelated to G6PD inactivation^30^. Besides, DHEA may enhance G6PD mRNA expression, confounding its inhibitory effects^31^. The lack of G6PD-specific inhibitors prompted us to explore alternative strategies to target G6PD and oxidative PPP. Recent studies have identified multiple regulators of G6PD at transcription and post-translational levels. For example, tumour suppressor p53 directly bound to and inhibited G6PD^17^. TAp73, a p53 family member, activates G6PD gene transcription^21^. Phosphatase and tensin homologue (PTEN) suppresses PPP pathway through abrogating G6PD pre-mRNA splicing and preventing the formation of active G6PD dimers^32^. Upon exposure to irradiation, ATM kinase promotes G6PD activity to enhance nucleotide production^33^. Post-translationally, G6PD is modified by lysine acetylation, phosphorylation, ubiquitination and O-GlcNAcylation^34^,^35^,^36^,^37^. We previously found that G6PD is deacetylated and activated by SIRT2^38^, which led us to explore the possibility of targeting SIRT2 to suppress G6PD and leukaemia^39^,^40^,^41^. SIRT2-mediated deacetylation of G6PD sustained NADPH production and promoted cell proliferation across different subtypes of AML. SIRT2-specific inhibitors effectively suppressed the proliferation of leukaemia cells, but not normal hematopoietic cells. The anti-proliferative effect of SIRT2 inhibitors merits further explorations in leukaemia mouse models and clinical trials. It is noteworthy that not every SIRT2 inhibitor achieved high efficacy of growth suppression in our cell proliferation assays. Development of more potent and specific inhibitors against SIRT2 is currently under active investigation^42^. Recently, it was proposed that SIRT2 inhibition may exhibit anti-cancer effect through promoting the degradation of Myc oncoprotein^43^. However, we found that shRNAs or chemical inhibitors against SIRT2 downregulated G6PD activity and suppressed leukaemia cell growth irrespective of Myc expression level (Fig. s1b). This observation indicates that the anti-cancer effect of SIRT2 inhibitors may be context-dependent.

Cancer cells consume large amounts of fatty acids for membrane production and lipid modification of proteins^44^. In the absence of G6PD, leukaemia cells exhibited enhanced demand for exogenous lipids, indicating that *G6PD*-knockdown cells are possibly deficient in *de novo* lipogenesis. Therefore, assimilating exogenous lipids would replenish lipid supplies and restore leukaemia cell proliferation. Supporting this notion, increased lipid mobilization had been observed in chronic lymphocytic leukaemia (CLL) patients as the lipolysis activity is significantly enhanced^45^,^46^.

G6PD depletion did not impair redox homeostasis of leukaemia cells, as GSH/GSSG ratio and ROS level remained unchanged in *G6PD*-knockdown cells. Ribose supplementation was incapable to rescue the growth defect of *G6PD*-knockdown cells. Yet it is possible that lack of G6PD affects ROS scavenging and ribose synthesis under stressed conditions, such as acute ROS challenge or DNA damage^47^,^48^.

Taken together, our study uncovers that leukaemia cells are dependent on oxidative PPP, in particular G6PD. SIRT2-mediated G6PD deacetylation and activation contributes to the development of AML and serves as a promising druggable target.

## Methods

### Generation of Stable Cell Pools

To generate stable knockdown cell pools in HL-60 cells, shRNA library containing two different shRNAs targeted towards each metabolic gene in pentose phosphate pathway were co-transfected with vectors expressing the *gag* and *vsvg* genes into HEK293T cells using a two-plasmid packaging system as previously described^49^,^50^. Retroviral supernatant was harvested 36 hrs after transfection, and mixed with 8 μg/mL polybrene to increase the infection efficiency. Cells were infected with the retrovirus and selected in 1 μg/ml puromycin for 1 week.

To generate stable *G6PD*- and/or *SIRT2*-knockdown cell pools in HL-60 and KG-1 cells, two different shRNAs targeting *SIRT2* gene were constructed in pMKO.1-hyg vector as described previously^51^ (sh*SIRT2*-#1: 5′-GCTCATCAACAAGGAGAAAGC-3′; sh*SIRT2*-#2: 5′-GCATGGACTTTGACTCCAAGA-3′). The virus was produced as described above. Retroviral supernatant was harvested 36 hrs after transfection, and mixed with 8 μg/mL polybrene to increase the infection efficiency. *G6PD*-knockdown stable cells were infected with the retrovirus and selected in 200 μg/ml hygromycin B for 1 week.

### Human Acute Myeloid Leukaemia Samples

The protocol of studies on human samples was approved by the Institutional Ethics Review Board of Southwest Hospital, Third Military Medical University. All methods were performed in accordance with the guidelines and regulations of Southwest Hospital, Third Military Medical University. Written informed consent was obtained from all participants. Leukaemia bone marrow samples were collected from AML patients. Control bone marrow samples were acquired from participants who have been excluded of haematological diseases. Mononuclear cells from these samples were prepared by red blood cell lyses method and were stored in liquid nitrogen until use. Bulk populations of normal or leukaemia cells were used for the analysis in this study.

### Statistical Analysis

Statistical analyses were performed with a two-tailed unpaired Student’s t-test. All data shown represent the results obtained from triplicate independent experiments with standard errors of the mean (mean ± S.D.). Kaplan-Meier survival curves were prepared using tumours with mRNA data (RNA Seq V2) (n = 173) from TCGA AML study. The patients were stratified by median mRNA expression Z-score. ‘High’ indicates greater than median (n = 86), ‘low’ denotes less than or equal to median (n = 86). P-value was determined by the log-rank test. The values of p < 0.05 were considered statistically significant.

## Additional Information

**How to cite this article**: Xu, S.-N. *et al*. SIRT2 activates G6PD to enhance NADPH production and promote leukemia cell proliferation. *Sci. Rep.* **6**, 32734; doi: 10.1038/srep32734 (2016).

## Supplementary Material

Supplementary Information
