# Supplementary material for: SIRT2 activates G6PD to enhance NADPH production and promote leukaemia cell proliferation
Source: Sci Rep. 2016 Sep 2;6:32734. doi: 10.1038/srep32734 (PMC5009355; doi:10.1038/srep32734)
Supplement: Supplementary Information [file srep32734-s1.pdf]

SIRT2 activates G6PD to enhance NADPH production and promote leukaemia cell proliferation

Shuang-Nian Xu[1, +,*], Tian-Shi Wang[2, +], Xi Li[1], and Yi-Ping Wang[2,*]

[1]Department of Haematology, Southwest Hospital, Third Military Medical University, Chongqing 400038, China.

[2]Department of Biochemistry and Molecular Cell Biology, Shanghai Key Laboratory for Tumour Microenvironment and Inflammation, Shanghai Jiao Tong University School of Medicine, 280 Chongqing South Rd., Shanghai 200025, China.

[+]these authors contributed equally to this work.

*Corresponding Author: Shuang-Nian Xu (email:xushuangnian1985@163.com); Yi-Ping Wang (email: wangyiping@shsmu.edu.cn);

a

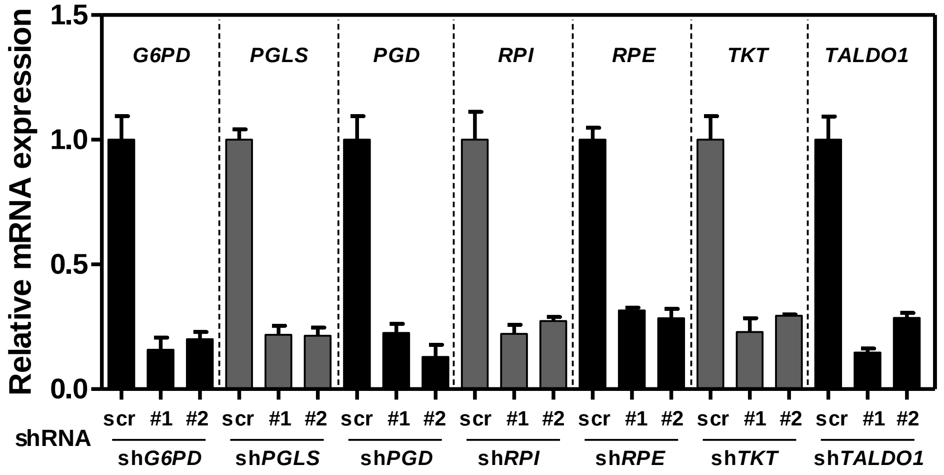

b

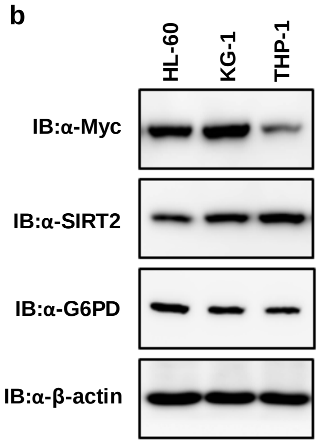

Fig.s1. (a) Knockdown efficiencies of shRNAs targeting each PPP genes were determined by qPCR. (b) Protein expression of Myc, SIRT2 and G6PD in HL-60, KG-1 and THP-1 cells were determined by western blotting, β-actin was included as a loading control.

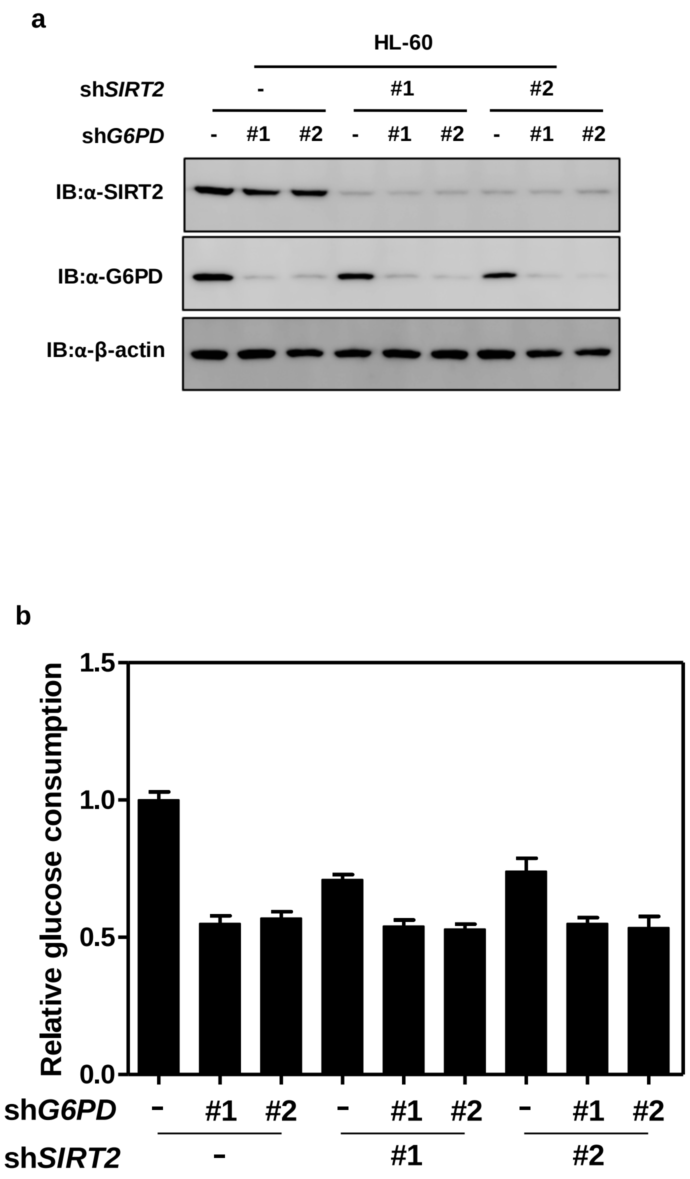

Fig.s2. (a) Knockdown efficiencies of shRNAs against *SIRT2* and/or *G6PD* were determined by western blotting, β-actin was included as a loading control. (b) Glucose consumption of HL-60 cells with stable knockdown of *SIRT2* and/or *G6PD* was determined.

a

Oxidative pentose phosphate pathway

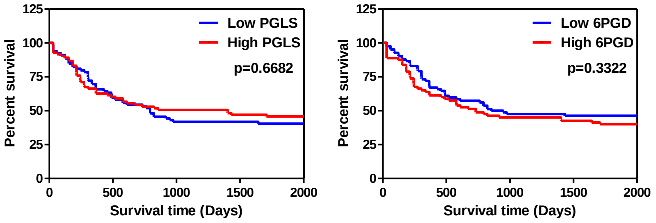

b

Non-oxidative pentose phosphate pathway

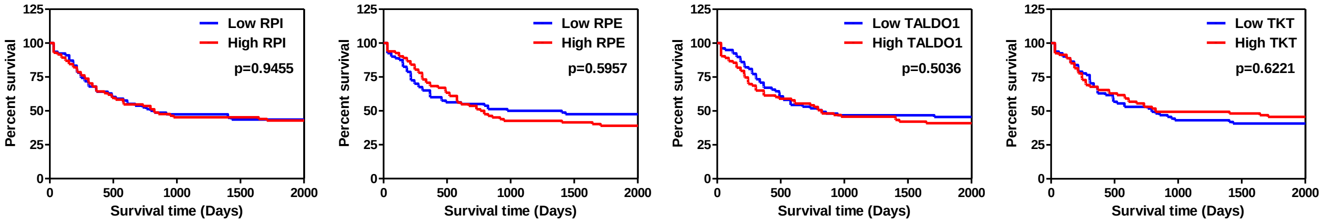

Fig.s3. Kaplan-Meier survival curves for TCGA AML study[1]. The TCGA AML patients (n=173) were stratified by median mRNA expression levels (Z-score adopted from cBioportal[2,3]) of enzymes in oxidative PPP (a) or non-oxidative PPP (b) as indicated ('high' is greater than median, 'low' is less than or equal to median).

Supplementary Table 1. Oligonucleotides used in this study

Name	Sequence	Note
**G6PD-Forward**	5'-CACCAGATGGTGGGGTAGAT-3'	qRT-PCR
**G6PD-Reverse**	5'-AGAGCTTTTCCAGGGCGAT-3'	qRT-PCR
**PGLS-Forward**	5'-AGCTCGGGGTTAATGGTGAT-3'	qRT-PCR
**PGLS-Reverse**	5'-CTTCGATCACGCCGAGAG-3'	qRT-PCR
**PGD-Forward**	5'-GCCTTGGAAGATGGTCTTGA-3'	qRT-PCR
**PGD-Reverse**	5'-GTCAGTGGTGGAGAGGAAGG-3'	qRT-PCR
**RPI-Forward**	5'-CGATCCAGATCACTGAGGGT-3'	qRT-PCR
**RPI-Reverse**	5'-GCTGAAAGGGTGAAGCAAGA-3'	qRT-PCR
**RPE-Forward**	5'-GGCTTTACCCACTGTTCTGG-3'	qRT-PCR
**RPE-Reverse**	5'-GGATGCTAGACTCTGGGGC-3'	qRT-PCR
**TKT-Forward**	5'-GCATGGTGTGGAAAAAGAGG-3'	qRT-PCR
**TKT-Reverse**	5'-CGCCTACGTATCAGCTCCA-3'	qRT-PCR
**TALDO1-Forward**	5'-ATCCTGGGGCTTGTACTCGT-3'	qRT-PCR
**TALDO1-Reverse**	5'-GAAGCGTCAGAGGATGGAGT-3'	qRT-PCR
**SIRT2-Forward**	5'-ATGTCTGCTTCTCCACCAGC-3'	qRT-PCR
**SIRT2-Reverse**	5'-GGTCGGTGACAGCCTCAAG-3'	qRT-PCR
**shG6PD-#1-Top**	5'-CCGGGCCTTCCATCAGTCGGATACACTCGAGTGTATCCGACTGATGGAAGGCTTTTTG-3'	shRNA cloning
**shG6PD-#1-Bottom**	5'-AATTCAAAAAGCCTTCCATCAGTCGGATACACTCGAGTGTATCCGACTGATGGAAGGC-3'	shRNA cloning
**shG6PD-#2-Top**	5'-CCGGGCAAACAGAGTGAGCCCTTCTCTCGAGAGAAGGGCTCACTCTGTTTGCTTTTTG-3'	shRNA cloning
**shG6PD-#2-Bottom**	5'-AATTCAAAAAGCAAACAGAGTGAGCCCTTCTCTCGAGAGAAGGGCTCACTCTGTTTGC-3'	shRNA cloning
**shPGLS-#1-Top**	5'-CCGGGCAGCACGAACTGTCATCTTTCTCGAGAAAGATGACAGTTCGTGCTGCTTTTTG-3'	shRNA cloning
**shPGLS-#1-Bottom**	5'-AATTCAAAAAGCAGCACGAACTGTCATCTTTCTCGAGAAAGATGACAGTTCGTGCTGC-3'	shRNA cloning
**shPGLS-#2-Top**	5'-CCGGGCACGAACTGTCATCTTTGTGCTCGAGCACAAAGATGACAGTTCGTGCTTTTTG-3'	shRNA cloning
**shPGLS-#2-Bottom**	5'-AATTCAAAAAGCACGAACTGTCATCTTTGTGCTCGAGCACAAAGATGACAGTTCGTGC-3'	shRNA cloning
**shPGD-#1-Top**	5'-CCGGGCTAGACTCATTCCTGATTGACTCGAGTCAATCAGGAATGAGTCTAGCTTTTTG-3'	shRNA cloning
**shPGD-#1-Bottom**	5'-AATTCAAAAAGCTAGACTCATTCCTGATTGACTCGAGTCAATCAGGAATGAGTCTAGC-3'	shRNA cloning
**shPGD-#2-Top**	5'-CCGGGGAAGACACACAGTTTATTTGCTCGAGCAAATAAACTGTGTGTCTTCCTTTTTG-3'	shRNA cloning
**shPGD-#2-Bottom**	5'-AATTCAAAAAGGAAGACACACAGTTTATTTGCTCGAGCAAATAAACTGTGTGTCTTCC-3'	shRNA cloning
**shRPI-#1-Top**	5'-CCGGGCTGATTTCAGGAAAGATTCGCTCGAGCGAATCTTTCCTGAAATCAGCTTTTTG-3'	shRNA cloning
**shRPI-#1-Bottom**	5'-AATTCAAAAAGCTGATTTCAGGAAAGATTCGCTCGAGCGAATCTTTCCTGAAATCAGC-3'	shRNA cloning
**shRPI-#2-Top**	5'-CCGGGGTCATCCCAATGGCCTATGTCTCGAGACATAGGCCATTGGGATGACCTTTTTG-3'	shRNA cloning
**shRPI-#2-Bottom**	5'-AATTCAAAAAGGTCATCCCAATGGCCTATGTCTCGAGACATAGGCCATTGGGATGACC-3'	shRNA cloning
**shRPE-#1-Top**	5'-CCGGATGCACATGATGGTGTCCAAGCTCGAGCTTGGACACCATCATGTGCATTTTTG-3'	shRNA cloning
**shRPE-#1-Bottom**	5'-AATTCAAAAAATGCACATGATGGTGTCCAAGCTCGAGCTTGGACACCATCATGTGCA-3'	shRNA cloning
**shRPE-#2-Top**	5'-CCGGTATAGAGGTCGATGGTGGAGTCTCGAGACTCCACCATCGACCTCTATATTTTTG-3'	shRNA cloning
**shRPE-#2-Bottom**	5'-AATTCAAAAATATAGAGGTCGATGGTGGAGTCTCGAGACTCCACCATCGACCTCTATA-3'	shRNA cloning
**shTKT-#1-Top**	5'-CCGGGCCGCCAATACAAAGGGTATCCTCGAGGATACCCTTTGTATTGGCGGCTTTTTG-3'	shRNA cloning
**shTKT-#1-Bottom**	5'-AATTCAAAAAGCCGCCAATACAAAGGGTATCCTCGAGGATACCCTTTGTATTGGCGGC-3'	shRNA cloning
**shTKT-#2-Top**	5'-CCGGGGAGGTTCACTTGCTTGTTGTCTCGAGACAACAAGCAAGTGAACCTCCTTTTTG-3'	shRNA cloning
**shTKT-#2-Bottom**	5'-AATTCAAAAAGGAGGTTCACTTGCTTGTTGTCTCGAGACAACAAGCAAGTGAACCTCC-3'	shRNA cloning
**shTALDO1-#1-Top**	5'-CCGGGCAAGGACCGAATTCTTATAACTCGAGTTATAAGAATTCGGTCCTTGCTTTTTG-3'	shRNA cloning

**shTALDO1-#1-Bottom**	5'-AATTCAAAAAGCAAGGACCGAATTCTTATAACTCGAGTTATAAGAATTCGGTCCTTGC-3'	shRNA cloning
**shTALDO1-#2-Top**	5'-CCGGGCAAACACCGACAAGAAATCCCTCGAGGGATTTCTTGTCGGTGTTTGCTTTTTG-3'	shRNA cloning
**shTALDO1-#2-Bottom**	5'-AATTCAAAAAGCAAACACCGACAAGAAATCCCTCGAGGGATTTCTTGTCGGTGTTTGC-3'	shRNA cloning
**shSIRT2-#1-Top**	5'-CCGGGCTCATCAACAAGGAGAAAGCCTCGAGGCTTTCTCCTTGTTGATGAGCTTTTTG-3'	shRNA cloning
**shSIRT2-#1-Bottom**	5'-AATTCAAAAAGCTCATCAACAAGGAGAAAGCCTCGAGGCTTTCTCCTTGTTGATGAGC-3'	shRNA cloning
**shSIRT2-#2-Top**	5'-CCGGGCATGGACTTTGACTCCAAGACTCGAGTCTTGGAGTCAAAGTCCATGCTTTTTG-3'	shRNA cloning
**shSIRT2-#2-Bottom**	5'-AATTCAAAAAGCATGGACTTTGACTCCAAGACTCGAGTCTTGGAGTCAAAGTCCATGC-3'	shRNA cloning

Supplementary Table 2. Characteristics of cell lines used in this study

Cell	Organism	Disease	FAB subtype	Cell type	Age (years)	Gender
**HL-60**	*Homo sapiens*	Acute promyelocytic leukemia	M2	Promyeloblast	36	Female
**THP-1**	*Homo sapiens*	Acute monocytic leukemia	M5	Monocyte	1	Male
**KG-1**	*Homo sapiens*	Acute myelogenous leukemia	M1	Macrophage	59	Male
**RAW 264.7**	*Mus musculus*	Abelson murine leukemia virus-induced tumor	N.A	Macrophage	N.A.	Male(BALB/c)

Supplementary Table 3. Chemical inhibitors used in this study

SIRT2 inhibitor	IC50	Specificity	Reference
**Sirtinol**	131μM	more potent against SIRT1 (IC50=38μM)	Grozinger CM *et al*.2001[4]
**AGK2**	3.5μM	Specific towards SIRT2	Ramakrishnan G *et al*.2014[5]
**SirReal2**	140nM	Specific towards SIRT2	Rumpf T *et al*.2015[6]

Supplementary Table 4. Characteristics of AML patients in this study

Patient Number	Age at diagnosis	Ethnicity	Gender	FAB subtype
02	53	Chinese	Female	AML-M2
03	30	Chinese	Male	AML-M3
13	30	Chinese	Male	AML-M3
14	27	Chinese	Female	AML-M4
78	46	Chinese	Female	AML-M4
84	49	Chinese	Female	AML-M5
97	52	Chinese	Female	AML-M6
98	67	Chinese	Female	AML-M4

Supplementary Methods

Reagents

Antibodies against G6PD (Abcam), SIRT2 (Abcam), β-actin (Cell Signaling) were purchased commercially. Dehydroepiandrosterone (DHEA, #709549), 6-aminonicotinamide (ANAD, #A68203), reduced glutathione (GSH, #G4251), N-acetyl cysteine (NAC, #1009005), myristic acid (MA, #M3128), palmitic acid (PA, #P5585), and stearic acid(SA, #175366) were obtained from Sigma. K403 site-specific antibody against acetylated G6PD [acG6PD(K403)] was obtained as previously described[7]. SIRT2 inhibitors, including Sirtinol (#S2804), AGK2 (#S7577), and SirReal2 (#S7845), were commercially purchased from Selleck. Ribonucleosides or deoxyribonucleosides were prepared by combining four ribonucleosides (Sigma, 200 μM) or four deoxyribonucleosides (Sigma, 200 μM).

Cell Culture

HL-60, KG-1, THP-1, and RAW 264.7 cells were maintained in RPMI-1640 medium supplemented with 10% heat-inactivated fetal bovine serum (MP Biomedicals), 2 mmol/L L-glutamine, 100 U/mL penicillin/streptomycin.

RNA Isolation and Quantitative Real-time PCR

Total RNA was isolated from cultured cells using Trizol reagent (Invitrogen) following the manufacturer's instructions. RNA was reverse transcribed with oligo-dT primers and preceded to real-time PCR with gene-specific primers in the presence of SYBR Premix Ex Taq (TaKaRa). β-actin was used as a housekeeping control.

Detection of Metabolites and Reactive Oxygen Species (ROS)

Concentrations of glucose 6-phosphate (G6P), 6-phosphogluconate (6PG), nicotinamide adenine dinucleotide phosphate ($NADPH/NADP^+$), glutathione (GSH/GSSG) were determined by using Glucose-6-Phosphate Assay Kit (Abcam, #ab83426), 6-Phosphogluconic Acid Assay Kit (Biovision, #K217-100), $NADP^+$/NADPH Assay Kit (Abcam, #ab65349), and Glutathione Assay Kit (Biovision, #K264-100) following the manufacturer's instructions.

ROS production was determined by using a fluorescent dye 2', 7'-dichlorofluorescein diacetate (H_2DCF-DA, Sigma). Briefly, 4×10^4 cells were washed with PBS and incubated with 10 μM H_2DCF-DA at 37°C for 30 min to load the fluorescent dye. Afterward, cells were washed with PBS twice. Fluorescence (Ex.488nm, Em.525nm) was monitored by a SpectraMax M5 Microplate Reader (Molecular Devices).

Cell Proliferation Assay

Cells were seeded in 6-well plates at 8,000 cells per well in 2 mL media. Plates were counted every day for 5 days or as indicated. For cell counting, leukaemia cells were centrifugized and re-suspended in PBS-EDTA, and counted by using a hemocytometer.

Cell Proliferation Assay by CCK-8

CCK-8 assay was carried out as previously described. Briefly, 100 μL of cells ($2x10^3$/ml) were seeded in triplicated wells of a 96-well microplate. After cells were cultured for 5 days, 10 μL of CCK-8 solution (Beyotime, China) was added to each well and incubated at 37 °C for 2 hrs. Plates were read on SpectraMax M5 Microplate Reader at 450 nm with a

reference wavelength at 630 nm. Relative cell proliferation (absorbance at 450 nm) was normalized against shscramble cells.

Colony Formation Assay

For colony formation assay, HL-60 cells were plated (1000 cells per 35mm dish) in methylcellulose-based MethoCult medium (Stem Cell Technologies). Culture dishes were incubated for 6 days at 37°C in a humid atmosphere with 5% CO_2. Colonies (>50 μm diameter) were counted using Olympus fluorescence microscope IX18.

Immunoprecipitation and Western Blotting

Cells were lysed in ice-cold NP-40 buffer [50 mM Tris-HCl (pH 7.6), 150 mM NaCl, 0.5% NP-40)] containing protease inhibitor cocktail (Roche). Immunoprecipitation was carried out by incubating G6PD antibody with cell lysate for 1 hr, followed by incubating with Protein-A beads (Upstate) for another 2 hrs at 4°C before beads were washed for three times with ice-cold NP-40 buffer. Standard western blot protocols were adopted. The quantification was carried out by subtracting background from the band intensity of western blots by using software ImageQuantTL (GE)

G6PD Enzyme Activity Assay

To obtain endogenous G6PD activity, both PGD activity alone and total dehydrogenase activity (G6PD+PGD) were measured separately. G6PD activity was calculated by subtracting the activity of PGD from total enzyme activity. Reaction mixture consists of 50 mM Tris-HCl (pH 7.6), 0.1 mM $NADP^+$, 0.2 mM glucose-6-phosphate (G6P) or 0.2 mM 6-phosphogluconate in a total volume of 300μL. Reactions were initiated by adding enzyme and analyzed at 25°C. Activities were measured by the conversion of $NADP^+$ to

NADPH, which was monitored by measuring the increase of fluorescence (Ex. 350nm, Em.470nm, HITACH F-4600 fluorescence spectrophotometer) for NADPH generation.

Glucose Uptake Assay

Glucose consumption was determined by using a colorimetric glucose assay kit (Abcam, #ab136955) following the manufacturer's instructions. Briefly, cells were seeded into 6-well plate at 4×10^5 per well. After 3hrs of cell culture, supernatant of the medium was collected subjected into glucose detection. The uptake of glucose was determined by subtracting the final medium glucose concentration from initial medium glucose concentration in the culture medium. Data was obtained from 3 independent triplicates and normalized against cell number.

Preparation of Hematopoietic Stem and Progenitor Cells (HSPCs)

Hematopoietic stem and progenitor cells were isolated as previously described[8]. Briefly, bone marrow cells were isolated from 6-8-week-old male BALB/c mice. Care of animals was in accordance with institutional guidelines. Whole bone marrow was obtained by flushing the femurs and tibias with an ice-cold solution of phosphate-buffered saline with 0.5% bovine serum albumin and 2 mM EDTA. Red blood cells were lysed using an RBC lysis solution (BioLegend, San Diego). A cell population enriched for HSPCs was obtained by magnetically depleting mature lineage-positive cells using antibodies to CD5, CD11b, CD45R, Ly-6G, and Ter119, according to the manufacturer's instructions (MAGM209, R&D Systems).

Supplementary References

1 Network, C. G. A. R. Genomic and epigenomic landscapes of adult de novo acute myeloid leukemia. N Engl J Med 368, 2059-2074, doi:10.1056/NEJMoa1301689 (2013).

2 Gao, J. et al. Integrative analysis of complex cancer genomics and clinical profiles using the cBioPortal. Sci Signal 6, pl1, doi:10.1126/scisignal.2004088 (2013).

3 Cerami, E. et al. The cBio cancer genomics portal: an open platform for exploring multidimensional cancer genomics data. Cancer Discov 2, 401-404, doi:10.1158/2159-8290.CD-12-0095 (2012).

4 Grozinger, C. M., Chao, E. D., Blackwell, H. E., Moazed, D. & Schreiber, S. L. Identification of a class of small molecule inhibitors of the sirtuin family of NAD-dependent deacetylases by phenotypic screening. J Biol Chem 276, 38837-38843, doi:10.1074/jbc.M106779200 (2001).

5 Ramakrishnan, G. et al. Sirt2 deacetylase is a novel AKT binding partner critical for AKT activation by insulin. J Biol Chem 289, 6054-6066, doi:10.1074/jbc.M113.537266 (2014).

6 Rumpf, T. et al. Selective Sirt2 inhibition by ligand-induced rearrangement of the active site. Nature communications 6, 6263, doi:10.1038/ncomms7263 (2015).

7 Wang, Y. P. et al. Regulation of G6PD acetylation by SIRT2 and KAT9 modulates NADPH homeostasis and cell survival during oxidative stress. The EMBO journal 33, 1304-1320, doi:10.1002/embj.201387224 (2014).

8 Field-Corbett, C., English, K. & O'Dea, S. Regulation of surfactant protein B gene expression in bone marrow-derived cells. Stem Cells 27, 662-669, doi:10.1634/stemcells.2008-0313 (2009).
